# Supplementary material for: Evaluation of a marker independent isolation method for circulating tumor cells in esophageal adenocarcinoma
Source: PLoS One. 2021 May 7;16(5):e0251052. doi: 10.1371/journal.pone.0251052 (PMC8104412; doi:10.1371/journal.pone.0251052)
Supplement: S1 Text — (DOCX) [file pone.0251052.s001.docx]

**S1 Text**

1. **Cell lines and cell culture**

The OE33 AND OE19 cell line are both from the ECACC cell lines (European Collection of Authenticated Cell Cultures). Cells were cultivated in DMEM with 10% FCS and 1% penicillin/streptomycin and grown in a 5% CO2 atmosphere at 37°C. Both cell lines were authenticated with short tandem repeat (STR) analysis. The cells were trypsinized and after centrifugation and washing steps adjusted to a cell suspension concentration in growth medium of 50 cells per μl. Next, 4μl of this suspension was spiked in the Streck and CellSave tubes containing whole blood of 9mL/tube from the healthy donors and in each of 4 wells of a 48 flat bottom well plate (Sigma-Aldrich) containing 100μl PBS. Directly after spike-in the blood tubes were slowly inverted and stored upright in the dark at room temperature. The well plate rested an hour on the benchtop after which the number of cancer cells on the bottom of each well was counted using a light microscope. The mean of these 4 counts was used to calculate the harvest rate where the number of cells counted on the glass slide after enrichment and manual staining was divided by the mean of the well-count as previously described.

1. **Patient selection**

Inclusion criteria were esophageal or gastro-esophageal junction adenocarcinoma (Siewert 1-3), clinical stage I - IV. The patient’s tumor was staged according to the most recent TNM classification for esophageal cancer (8th edition of AJCC/UICC staging of cancers of the esophagus and esophagogastric junction) [1]. Locally advanced tumors were treated with neoadjuvant chemotherapy or chemoradiation. The therapy regimes were CROSS (weekly administration of five cycles of neoadjuvant chemoradiotherapy (intravenous carboplatin [AUC 2 mg/mL per min] and intravenous paclitaxel [50 mg/m2 of body-surface area] for 23 days) with concurrent radiotherapy (41,4 Gy, given in 23 fractions of 1,8 Gy on 5 days per week))[2], FLOT (docetaxel, oxaliplatin and 5-fluorouracil) [3] and modified CROSS (mCROSS) (total radiation dose of 45 Gy). One patient diagnosed with metastatic disease was treated with Cisplatinum-5FU and radiation therapy with a total dose of 30 Gy. Follow-up data were last updated on November 25^th^, 2020.

1. **Blood sampling for healthy donors**

Thirty-six peripheral blood samples were collected from 8 healthy blood donors. An EDTA discard tube of 6ml, 2 CellSave Preservative tube of 9ml (Menarini) and 2 Streck Cell-free DNA BCT Streck tube of 10ml were filled with 9mL of venous blood [4, 5]. The EDTA tube was discarded because elimination of dead space in the butterfly needle set but also to avoid potential contamination of epithelial skin cells. The other tubes were gently inverted 10 times immediately after venipuncture and kept in upright position at room temperature according to the user instructions of the preservation tubes. Per cell line and for every enrichment technique a negative control was included. Consequently, experiment number 4 and 8 have each 2 samples extra, 1 CellSave tube and 1 Streck tube where we spiked no tumoral cells into.

Two different esophageal adenocarcinoma cell lines (OE33 and OE19) were used to spike an average of 200 cells per blood sample (mean ±SD number of cells spiked: 200±28).

Per experiment, the 2 Streck tubes were analyzed using the Parsortix workflow, the 2 CellSave tubes were analyzed using the CellSearch workflow. The technique and procedure of blood sampling was the same for patients and healthy donors. An overview of the experimental set-up is given in **S1 table**.

1. **Immunofluorescence staining**

During the whole procedure, LoRetention Dualfilter Eppendorf tips were used. After removing the supernatant, 90 µl autoMACS running buffer (Miltenyi Biotec 030-091-221) was added on top along with 3 µl anti CD45-APC (Human, Clone REA747 Miltenyi Biotec 130-110-633). This was vortexed slowly and incubated 20 min on ice at 4°C and in the dark. After incubation, 1ml autoMACS running buffer was added and centrifuged at 400G 10 min at 4°C. After removing supernatant, 90 µl Inside Perm (Inside stain kit, Miltenyi Biotec 130-091-221) was added on top together with 5 µl CK-FITC (conjugated antibody, firm), vortexed slow and incubated on ice 20 min in the dark. After incubation, 1 ml Inside perm was added on top and the sample was centrifuged on 400 G 10 min 4°C. The supernatant was removed. 1ml of 0.075M KCL buffer was added together with 1 µl Hoechst (Hoechst 33342, 1mg/ml) and vortexed slowly. After 5 min incubation on room temperature in the dark the sample was again centrifuged on 400 G 10 min on room temperature. Supernatant was removed without disturbing the pellet until an estimated 20 µl remained in the Eppendorf tube. This protocol was adapted from the immunofluorescence staining protocol for fixed cells in suspension provided by Menarini Silicon Biosystems (version 1.3).

**S1 Table. Experiment overview with number of enriched tumor cells and recovery ratio’s post Parsortix and CellSearch in spiked donor blood samples with OE33 and OE19 esophageal cancer cell lines.**

**S1 Table:** N: Number, Exp: Experiment, Cell line: oesophageal adenocarcinoma cell line OE33 or OE19, Harvest rate is calculated as N cells found/N cells spiked, Negative controls showed no tumor cells.

**S2 Table. Summary of diameter statistics (μm)**

**A Cell diameter**

| **Cell line** | **Cell type** | **Enrichment system** | **Number of data** | **Average** | **Median** | **Standard deviation** | **Minimum** | **Maximum** |
| --- | --- | --- | --- | --- | --- | --- | --- | --- |
| OE19 | CTC | CellSearch | 53 | 13.64 | 13.13 | 1.81 | 9.49 | 18.8 |
| OE19 | CTC | Parsortix | 165 | 14.05 | 13.82 | 1.7 | 10.51 | 22.46 |
| OE19 | WBC | CellSearch | 118 | 10.64 | 10.57 | 1.32 | 6.92 | 15.73 |
| OE19 | WBC | Parsortix | 993 | 11.03 | 10.79 | 1.38 | 6.96 | 16.48 |
| OE33 | CTC | CellSearch | 54 | 13.58 | 13.3 | 1.74 | 9.91 | 18.01 |
| OE33 | CTC | Parsortix | 477 | 16.16 | 16.18 | 1.73 | 10.79 | 22.6 |
| OE33 | WBC | CellSearch | 114 | 10.34 | 10.31 | 0.96 | 8.33 | 12.95 |
| OE33 | WBC | Parsortix | 297 | 11.63 | 10.69 | 2.26 | 8.72 | 17.55 |
| Both | CTC | CellSearch | 107 | 13.61 | 13.22 | 1.77 | 9.49 | 18.8 |
| Both | CTC | Parsortix | 642 | 15.62 | 15.6 | 1.95 | 10.51 | 22.6 |
| Both | WBC | CellSearch | 232 | 10.49 | 10.39 | 1.16 | 6.92 | 15.73 |
| Both | WBC | Parsortix | 1290 | 11.17 | 10.76 | 1.65 | 6.96 | 17.55 |

**B Nucleus diameter**

| **Cell line** | **Cell type** | **Enrichment system** | **Number of data** | **Average** | **Median** | **Standard deviation** | **Minimum** | **Maximum** |
| --- | --- | --- | --- | --- | --- | --- | --- | --- |
| OE19 | CTC | CellSearch | 53 | 10.45 | 10.43 | 1.62 | 7.15 | 14.99 |
| OE19 | CTC | Parsortix | 165 | 11.25 | 11.11 | 1.26 | 8.63 | 17.19 |
| OE19 | WBC | CellSearch | 118 | 8.89 | 8.75 | 1.35 | 6.23 | 13.53 |
| OE19 | WBC | Parsortix | 993 | 9.59 | 9.34 | 1.19 | 6.96 | 14.47 |
| OE33 | CTC | CellSearch | 54 | 10.34 | 10.52 | 1.65 | 6.37 | 13.69 |
| OE33 | CTC | Parsortix | 477 | 13.19 | 13 | 1.6 | 7.96 | 17.56 |
| OE33 | WBC | CellSearch | 114 | 8.54 | 8.4 | 1.1 | 6.37 | 11.32 |
| OE33 | WBC | Parsortix | 297 | 10.11 | 9.14 | 2.42 | 6.96 | 17.39 |
| Both | CTC | CellSearch | 107 | 10.4 | 10.5 | 1.63 | 6.37 | 14.99 |
| Both | CTC | Parsortix | 642 | 12.69 | 12.5 | 1.74 | 7.96 | 17.56 |
| Both | WBC | CellSearch | 232 | 8.72 | 8.6 | 1.24 | 6.23 | 13.53 |
| Both | WBC | Parsortix | 1290 | 9.71 | 9.33 | 1.58 | 6.96 | 17.39 |

**S3 Table. Literature overview of CTCs in Esophageal Adenocarcinoma**

| **Reference** | **Country** | **N** | **Population (N stage I-II/ N stage III-IVA/ N IVB)** | **Detection method** | **Volume blood sample** | **Cut-off £** | **CTC positivity rate % (n/N)** | **Stage (N CTC+)** |
| --- | --- | --- | --- | --- | --- | --- | --- | --- |
| Woestemeier et al. [6], 2018 | Germany | 32 | EAC, Stage NS | CellSearch | 7,5 mL | ≥1 CTCs | 18,8% (6/32) | NS |
| Woestemeier et al. [7], 2020 | Germany | 90 | EAC (60), SCC (27), Stage (42/44/4) | 1. IME (positive selection for CK and EPCaM) 2. Immunocytochemical staining (CD45, CK) | 7,5 mL | ≥1 CTCs | 25,6% (23/90) | NS |
| Kuvendjiska et al. [8], 2019 | Germany | 20 | EAC (0/20/0) | 1. Screencell Cyto Kit 2. May-Grünwald Giemsa staining | 6 mL | ≥1 CTCs | 30% (6/20) | NS |
| Kuvendjiska et al. [9], 2020 | Germany | 117 | Non-metastatic EAC, Stage NS | 1. Screencell Cyto Kit 2. May-Grünwald Giemsa staining | 6 mL | ≥1 CTCs | 69,2% (81/117) | NS |
| Reeh et al. [10], 2015 | Germany | 68 | EAC (32/32/4) | CellSearch | 7,5 mL | ≥1 CTCs | 20,6% (14/68) | StageI-II (5), Stage III-IVA (6), Stage IVB (3) |
| Kubisch et al. [11], 2015 | Germany | 62 | Advanced GC (39), EAC (25), Stage III (4), Stage IV (58) | 1. IME (positive selection for EPCaM and MUC-1) 2. RT-PCR for 5 different genes | 10 mL | ≥1 gene* | 69,4% (43/62) | NS |
| Sclafani et al. [12], 2014 | UK | 18 | EAC (2), GEJC (9), GC (7) Stage NS | CellSearch | 7,5 mL | ≥2 CTCs | 44,4 % (8/18) | EAC (0), GEJC (4), GC (4) |
| Wang et al. [13], 2016 | China | 78 | EAC (n=11) SCC (n= 67) Stage NS | 1. IME (positive selection for EPCaM) 2. Flow cytometry (CD45, CK) | 8 mL | ≥1 CTCs | 6,4% (5/78) | NS |
| Piegeler et al. [14], 2016 | Switzerland | 8 | EAC (0/6/2) | CellSearch | 7,5 mL | ≥1 CTCs | 12,5% (1/8) | NS |
| Bobek et al. [15], 2014 | Czech Republic | 20 | EAC (4/14/2) | Metacell | 8 mL | ≥1 CTCs | 75% (15/20) | Stage I-II (3), Stage III-IV (11), MD (1) |
| Pernot et al. [16], 2017 | France | 106 | EC (21), GC (54), GEJC (31), All Stage III-IV | CellSearch | 7,5 mL | ≥1 CTCs | 62% (66/106) | NS |
| Dent et al. [17], 2016 | UK | 6 | EAC (2/4/0) | 1. IME (negative selection for CD45-depletion) 2. Flow cytometry (positive selection for EPCaM +, CD45-) | 12 mL | ≥1 CTCs | 33,3% (2/6) | Stage III (2) |

**S3 Table:** All studies included were of prospective design. All blood samples were taken before any treatment was given. N: Number, EAC: Esophageal adenocarcinoma, SCC: squamous cell carcinoma, EC: esophageal cancer, GC: Gastric Cancer, GEJC: Gastro-Esophageal Junction Cancer, NS: Not specified in study, IME: Immunomagnetic enrichment where magnetic beads links to a marker are used to perform magnetic cell separation eg EPCaM, CK: cytokeratin, RT-PCR: Reverse transcription polymerase chain reaction, *: 1 gene tested positive (KRT19, MUC-1, EpCAM, CEACAM5, BIRC5) is counted as CTC positive, ¥: cell sorting with flow cytometry-technology using immunofluorescence antibodies cytokeratin and CD-45, £: cut-off value for being labeled as CTC-positive.

**S1 Fig. Images for identified CTCs per enrichment platform**

1. Enriched OE19-cells with Parsortix on glass slide (IF microscope ZEISS)

| 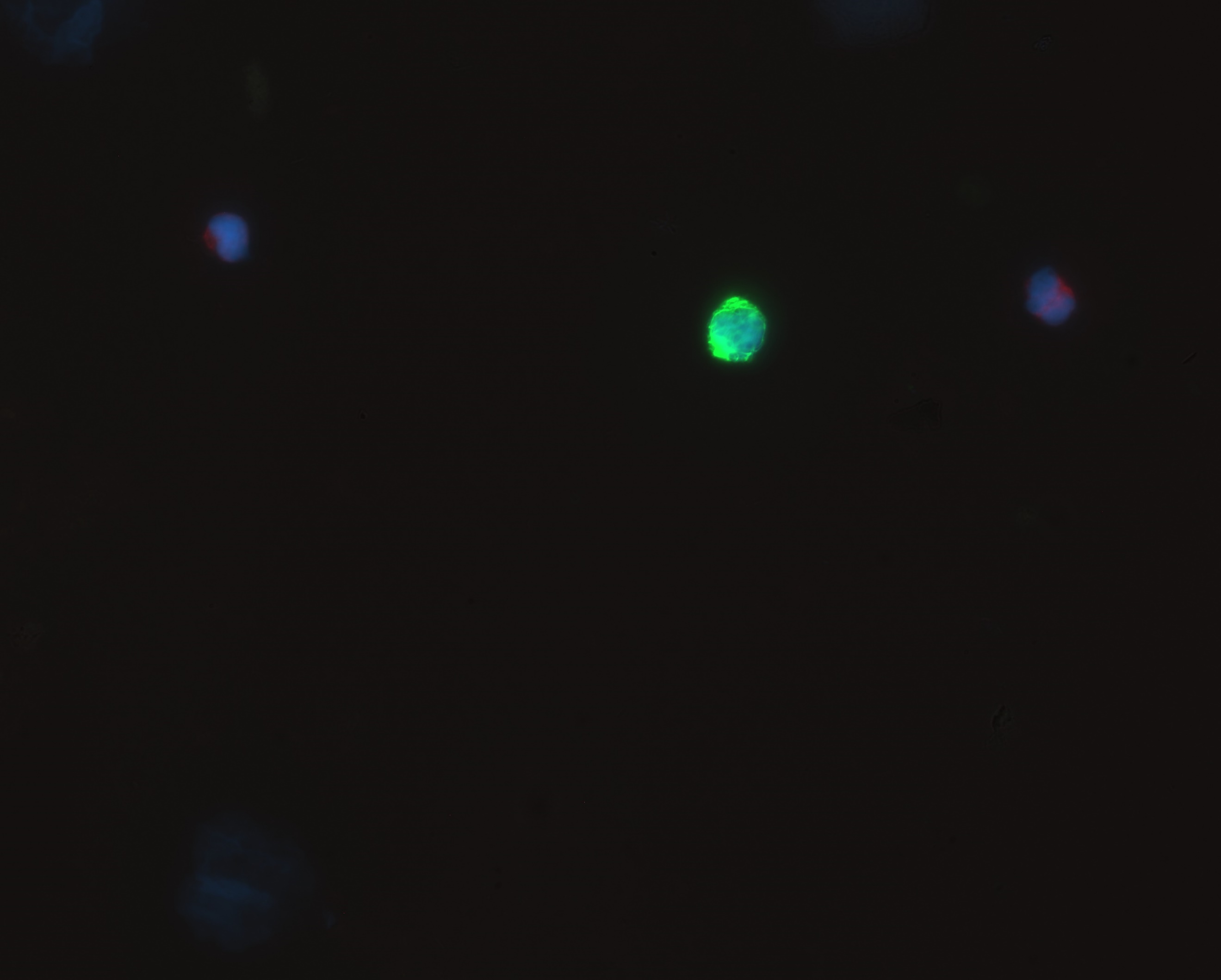 | 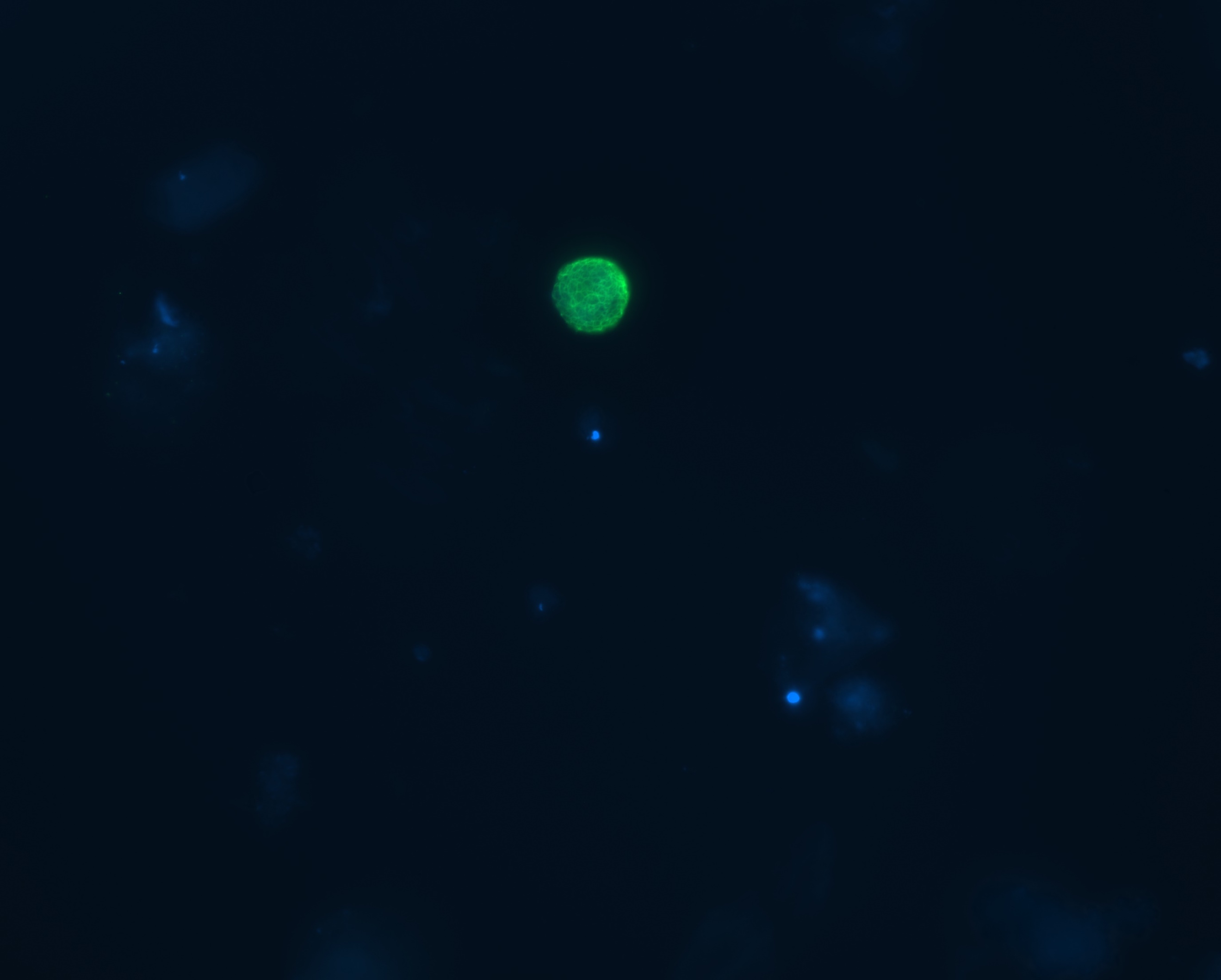 | 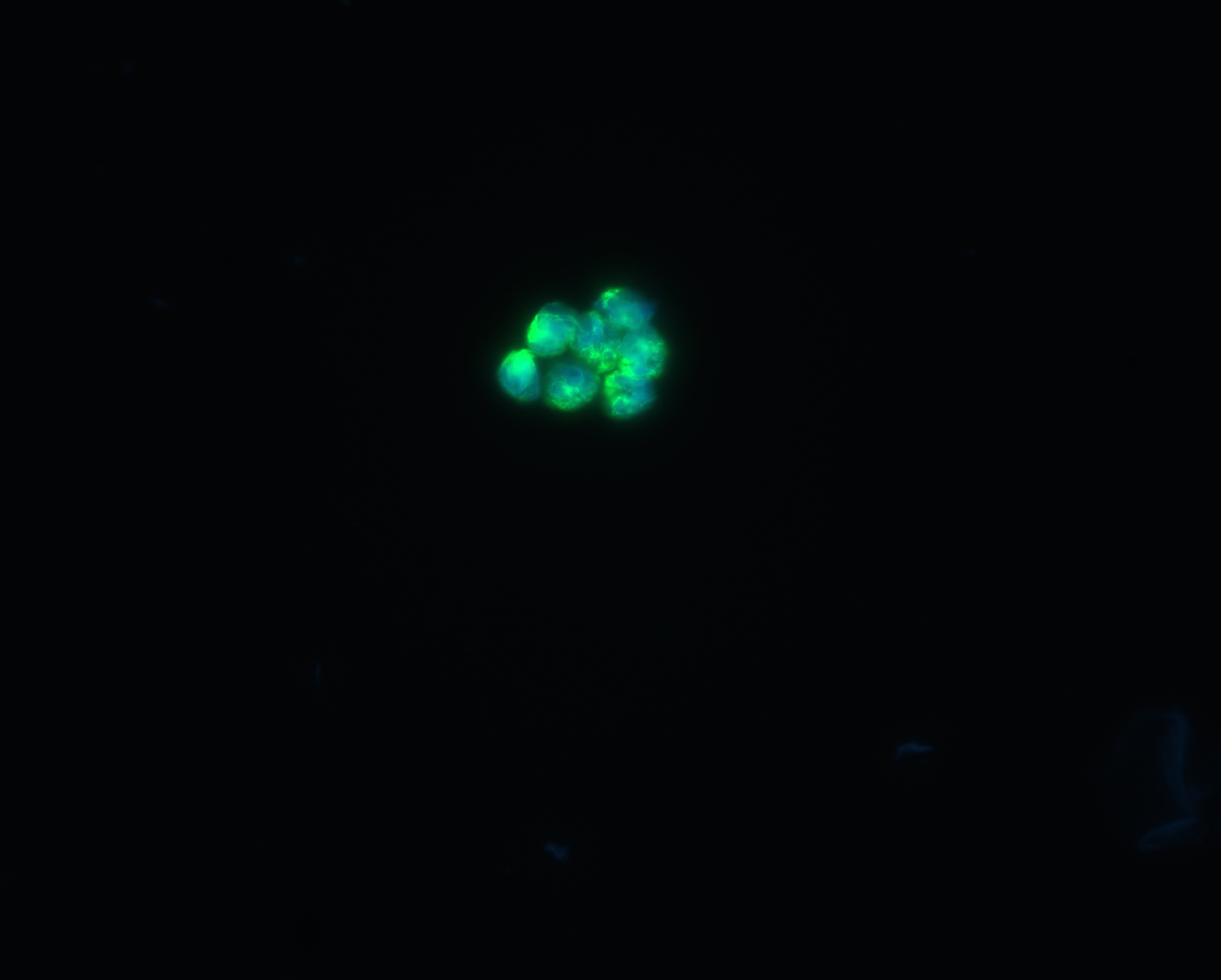 |
| --- | --- | --- |

1. Enriched OE33-cells with Parsortix on glass slide (IF microscope ZEISS)

| 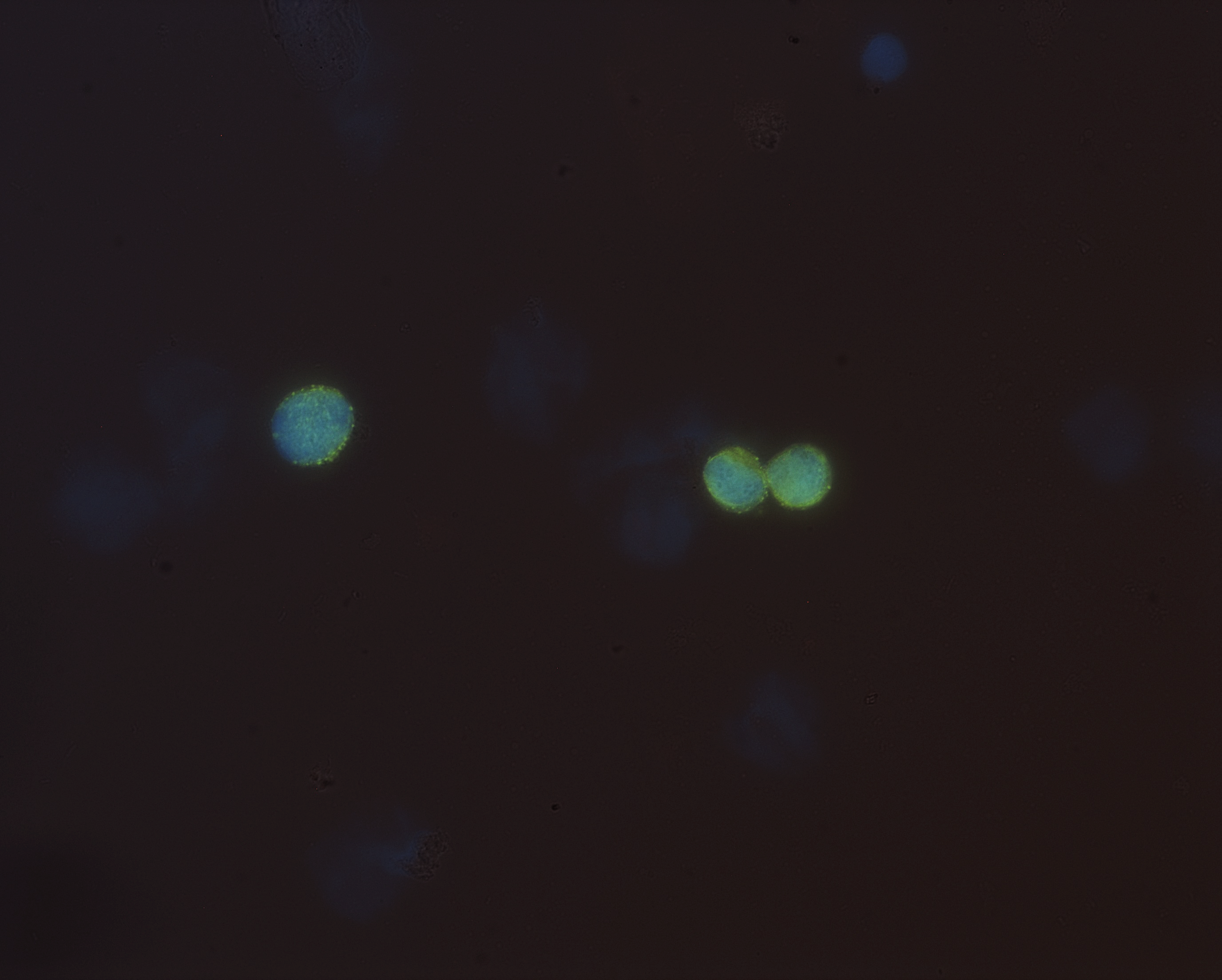 | 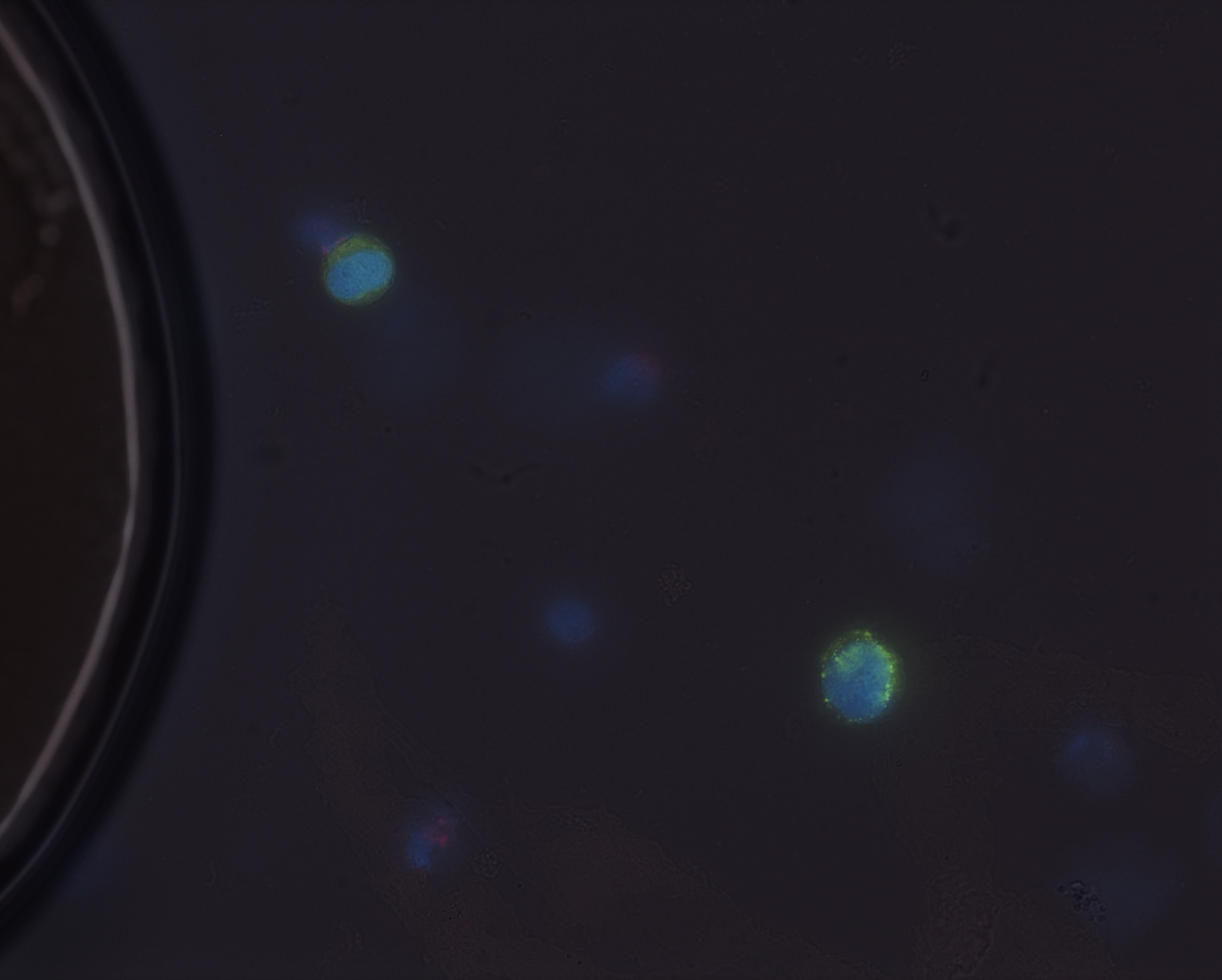 | 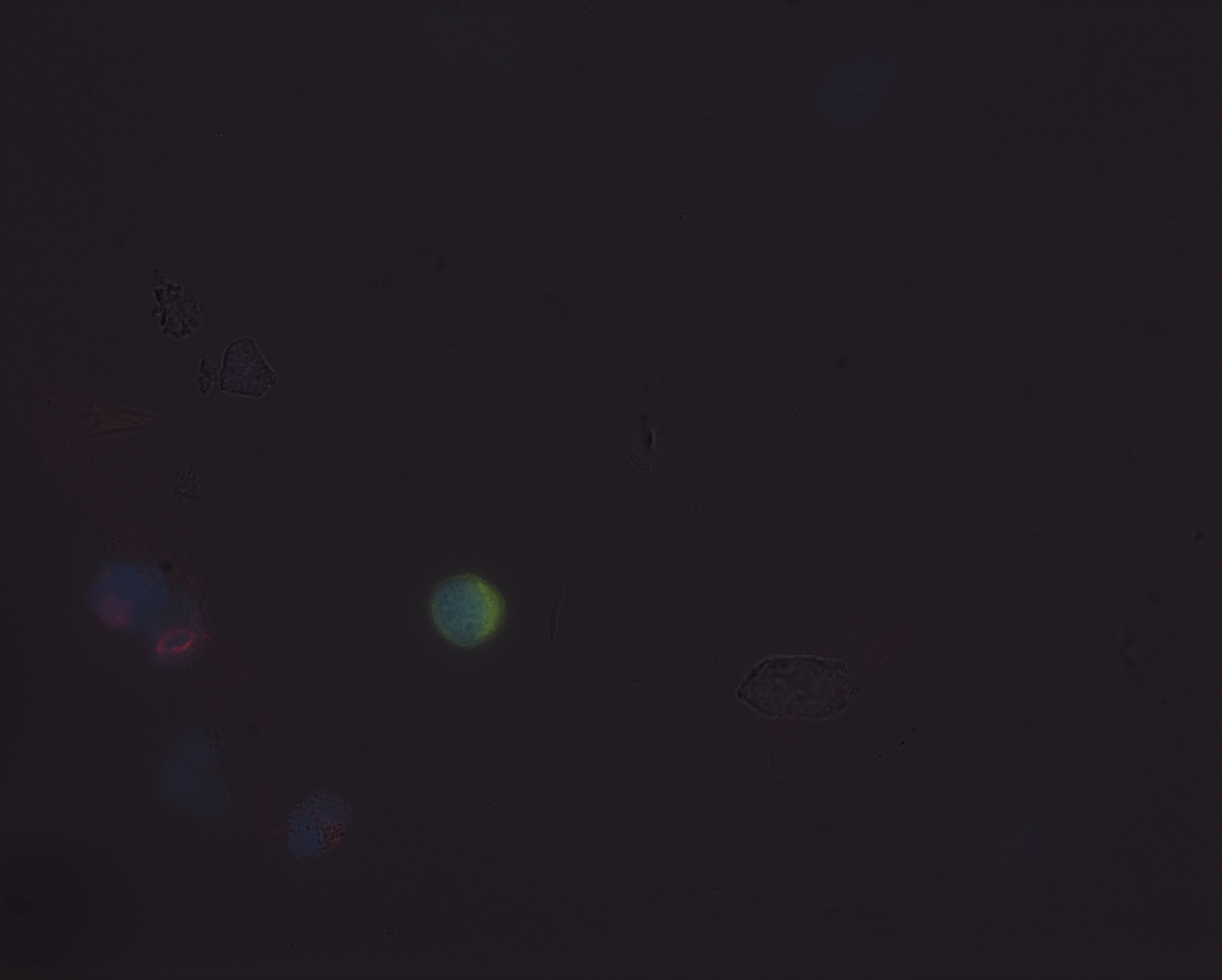 |
| --- | --- | --- |

1. Enriched tumor cells with CellSearch

|  | DAPI/CK-PE | CK-PE | DAPI | CD45-APC |
| --- | --- | --- | --- | --- |
| OE19 | 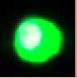 | 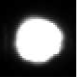 | 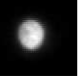 | 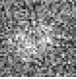 |
| OE33 | 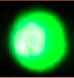 | 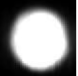 | 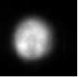 | 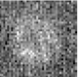 |

**S1 Fig:** A and b: Immunofluorescence microscope showing a CTC in a spiked healthy donor blood sample with an irregular and larger cell nucleus (Hoechst nucleus staining (blue)) and in comparison lower volume of cell membrane (FITC-CK (green)). Staining for CD45-APC (red) was negative. C shows an image gallery of CTCs. All immunofluorescence channels are depicted above (DAPI/CK-PE, CK-PE, DAPI and CD45-APC).

**S2 Fig. Representative images for identified CTCs on DEPArray Nxt**

| Cell line | Enrichment | CD45-APC | CK-FITC | Hoechst | Brightfield |
| --- | --- | --- | --- | --- | --- |
| OE19 | Parsortix | 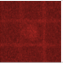 | 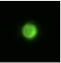 | 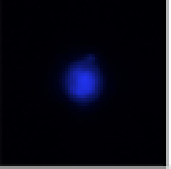 | 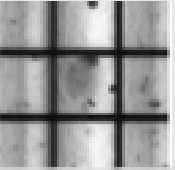 |
| OE19 | CellSearch | 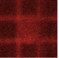 | 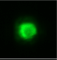 | 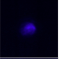 | 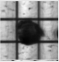 |
| OE33 | Parsortix | 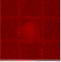 | 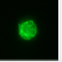 | 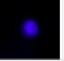 | 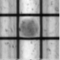 |
| OE33 | CellSearch | 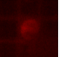 | 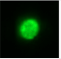 | 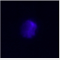 | 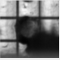 |

**S2 Fig:** Image gallery of selected CTC per enrichment platform. All immunofluorescence channels above (CD45-APC, CK-FITC, Hoechst). Brightfield is a photographic picture taken of the cell in the dielectrophoresis cages.

**References**

1. Rice TW, Patil DT, Blackstone EH. 8th edition AJCC/UICC staging of cancers of the esophagus and esophagogastric junction: application to clinical practice. Ann Cardiothorac Surg. 2017;6(2):119-30.
2. Shapiro J, van Lanschot JJB, Hulshof M, van Hagen P, van Berge Henegouwen MI, Wijnhoven BPL, et al. Neoadjuvant chemoradiotherapy plus surgery versus surgery alone for oesophageal or junctional cancer (CROSS): long-term results of a randomised controlled trial. Lancet Oncol. 2015;16(9):1090-8.
3. Al-Batran SE, Hofheinz RD, Pauligk C, Kopp HG, Haag GM, Luley KB, et al. Histopathological regression after neoadjuvant docetaxel, oxaliplatin, fluorouracil, and leucovorin versus epirubicin, cisplatin, and fluorouracil or capecitabine in patients with resectable gastric or gastro-oesophageal junction adenocarcinoma (FLOT4-AIO): results from the phase 2 part of a multicentre, open-label, randomised phase 2/3 trial. Lancet Oncol. 2016;17(12):1697-708.
4. Chudziak J, Burt DJ, Mohan S, Rothwell DG, Mesquita B, Antonello J, et al. Clinical evaluation of a novel microfluidic device for epitope-independent enrichment of circulating tumour cells in patients with small cell lung cancer. Analyst. 2016;141(2):669-78.
5. Norton SE, Lechner JM, Williams T, Fernando MR. A stabilizing reagent prevents cell-free DNA contamination by cellular DNA in plasma during blood sample storage and shipping as determined by digital PCR. Clin Biochem. 2013;46(15):1561-5.
6. Woestemeier A, Ghadban T, Riethdorf S, Harms-Effenberger K, Konczalla L, Uzunoglu FG, et al. Absence of HER2 Expression of Circulating Tumor Cells in Patients with Non-Metastatic Esophageal Cancer. Anticancer research. 2018;38(10):5665-9.
7. Woestemeier A, Harms-Effenberger K, Karstens KF, Konczalla L, Ghadban T, Uzunoglu FG, et al. Clinical Relevance of Circulating Tumor Cells in Esophageal Cancer Detected by a Combined MACS Enrichment Method. Cancers (Basel). 2020;12(3).
8. Kuvendjiska J, Bronsert P, Martini V, Lang S, Pitman MB, Hoeppner J, et al. Non-Metastatic Esophageal Adenocarcinoma: Circulating Tumor Cells in the Course of Multimodal Tumor Treatment. Cancers (Basel). 2019;11(3).
9. Kuvendjiska J, Pitman MB, Martini V, Braun C, Grebe K, Timme S, et al. Cytopathological Heterogeneity of Circulating Tumor Cells in Non-metastatic Esophageal Adenocarcinoma. Anticancer Res. 2020;40(10):5679-85.
10. Reeh M, Effenberger KE, Koenig AM, Riethdorf S, Eichstadt D, Vettorazzi E, et al. Circulating Tumor Cells as a Biomarker for Preoperative Prognostic Staging in Patients With Esophageal Cancer. Annals of surgery. 2015;261(6):1124-30.
11. Kubisch I, de Albuquerque A, Schuppan D, Kaul S, Schaich M, Stolzel U. Prognostic Role of a Multimarker Analysis of Circulating Tumor Cells in Advanced Gastric and Gastroesophageal Adenocarcinomas. Oncology. 2015;89(5):294-303.
12. Sclafani F, Smyth E, Cunningham D, Chau I, Turner A, Watkins D. A pilot study assessing the incidence and clinical significance of circulating tumor cells in esophagogastric cancers. Clin Colorectal Cancer. 2014;13(2):94-9.
13. Wang HB, Guo Q, Li YH, Sun ZQ, Li TT, Zhang WX, et al. Effects of Minimally Invasive Esophagectomy and Open Esophagectomy on Circulating Tumor Cell Level in Elderly Patients with Esophageal Cancer. World J Surg. 2016;40(7):1655-62.
14. Piegeler T, Winder T, Kern S, Pestalozzi B, Schneider PM, Beck-Schimmer B. Detection of circulating tumor cells in patients with esophagogastric or pancreatic adenocarcinoma using the CellSearch(R) system: An observational feasibility study. Oncology letters. 2016;12(2):1513-8.
15. Bobek V, Matkowski R, Gurlich R, Grabowski K, Szelachowska J, Lischke R, et al. Cultivation of circulating tumor cells in esophageal cancer. Folia Histochem Cytobiol. 2014;52(3):171-7.
16. Pernot S, Badoual C, Terme M, Castan F, Cazes A, Bouche O, et al. Dynamic evaluation of circulating tumour cells in patients with advanced gastric and oesogastric junction adenocarcinoma: Prognostic value and early assessment of therapeutic effects. Eur J Cancer. 2017;79:15-22.
17. Dent BM, Ogle LF, O'Donnell RL, Hayes N, Malik U, Curtin NJ, et al. High-resolution imaging for the detection and characterisation of circulating tumour cells from patients with oesophageal, hepatocellular, thyroid and ovarian cancers. Int J Cancer. 2016;138(1):206-16.
