# Supplementary material for: Evaluation of a marker independent isolation method for circulating tumor cells in esophageal adenocarcinoma
Source: PLoS One. 2021 May 7;16(5):e0251052. doi: 10.1371/journal.pone.0251052 (PMC8104412; doi:10.1371/journal.pone.0251052)
Supplement: Attachment — Submitted filename: Response to Reviewers.docx [file pone.0251052.s002.docx]

Object: Rebuttal letter

*March 25th, 2021*

Dear Editor,

We were pleased to have an opportunity to revise our manuscript now entitled “EVALUATION OF A MARKER INDEPENDENT ISOLATION METHOD FOR CIRCULATING TUMOR CELLS IN ESOPHAGEAL ADENOCARCINOMA” In the revised manuscript, we have carefully considered the reviewers’ comments and suggestions. As instructed, we have attempted to succinctly explain changes made in reaction to all comments. We reply to each comment in point-by-point fashion.

The reviewers’ comments were very helpful overall, and we are appreciative of such constructive feedback on our original submission.

Sincerely,

Annouck Philippron

**Comments**

Reviewer #1:

1.

**Reviewer:** Authors aim attention at first to compare the gold standard Cellsearch and Parsortix technologies to enrich putative CTCs (OE33 and OE19 cell lines). Authors found that CellSearch had a higher capture rate instead of Parsortix, but they chose to perform CTCs enrichment on esophageal cancer patients only by the Parsortix platform, Authors should explain better the reason for this choice.

**Authors:** We chose the Parsortix platform to be evaluated, benchmarked against the Cellsearch platform. After completing the comparison between the two platforms using esophageal cell lines, we continued with Parsortix only on patient samples. We did not take extra blood samples for analysis on Cellsearch because this was not the goal set forward in this study.

2.

**Reviewer:** Authors performed a morphology analysis of putative CTCs though DEPArray technology, but it is not clear why Authors did not use morphology analysis for patient samples. This is a matter of concern since authors often highlighted the potential usefulness of CTC analysis as well as molecular characterization; but they did not perform any type of this kind of analysis. More specifically Lines: 25 “the molecular characterization of circulating tumor cells (CTCs)...” and Line 39 “aiming towards downstream single-cell molecular characterization” authors stated these on the abstract objective and methods sections, but they did not perform this analysis; this kind of assumption should be addressed only in the discussion section this point is misleading.

**Authors:** We realise this can be misleading. We did not perform the same analysis with DEPArray on the patient samples because the number of CTC’s per blood tube from an esophageal cancer patient in currative setting is simply too few. Future aspects in this study are that a higher CTC prevalence can be found in metastatic patients which can make CTC analysis- including molecular characterization- possible. We will adapt all reference to ‘molecular characterization’ in the manuscript to avoid misinterpretation and add this aspect to future perspectives at the end in the section ‘Discussion’.

3.

**Reviewer:** Overall this manuscript shed a tiny light on the CTCs analysis in blood of esophageal cancer patients, but authors should pay attention to do not overstate their findings, I referred especially with DEPArray analysis which was not performed on blood patients and molecular analysis not performed as well, descriptive manuscript, carefull to not overstate findings, we carefully reviewed this manuscript

**Authors:** We agree with the reviewer and will revise this descriptive manuscript to not overstate our findings. However, we were carfull to write a ‘descriptive’ manuscript and to not formulate statements because of the more explorative study with low number of patient samples.
